# Intracellular Interactions Between Arboviruses and *Wolbachia* in *Aedes aegypti*

**DOI:** 10.3389/fcimb.2021.690087

**Published:** 2021-06-23

**Authors:** Jerica Isabel L. Reyes, Yasutsugu Suzuki, Thaddeus Carvajal, Maria Nilda M. Muñoz, Kozo Watanabe

**Affiliations:** ^1^ Center for Marine Environmental Studies (CMES), Ehime University, Matsuyama, Japan; ^2^ Graduate School of Science and Engineering, Ehime University, Matsuyama, Japan; ^3^ Biological Control Research Unit, Center for Natural Sciences and Environmental Research (CENSER), De La Salle University, Metro Manila, Philippines; ^4^ Research and Development Extension, Cagayan State University, Tuguegarao City, Philippines

**Keywords:** *Aedes aegypti*, dengue, zika, chikungunya, *Wolbachia*, vector control

## Abstract

*Aedes aegypti* is inherently susceptible to arboviruses. The geographical expansion of this vector host species has led to the persistence of Dengue, Zika, and Chikungunya human infections. These viruses take advantage of the mosquito’s cell to create an environment conducive for their growth. Arboviral infection triggers transcriptomic and protein dysregulation in *Ae. aegypti* and in effect, host antiviral mechanisms are compromised. Currently, there are no existing vaccines able to protect human hosts from these infections and thus, vector control strategies such as *Wolbachia* mass release program is regarded as a viable option. Considerable evidence demonstrates how the presence of *Wolbachia* interferes with arboviruses by decreasing host cytoskeletal proteins and lipids essential for arboviral infection. Also, *Wolbachia* strengthens host immunity, cellular regeneration and causes the expression of microRNAs which could potentially be involved in virus inhibition. However, variation in the magnitude of *Wolbachia*’s pathogen blocking effect that is not due to the endosymbiont’s density has been recently reported. Furthermore, the cellular mechanisms involved in this phenotype differs depending on *Wolbachia* strain and host species. This prompts the need to explore the cellular interactions between *Ae. aegypti*-arboviruses-*Wolbachia* and how different *Wolbachia* strains overall affect the mosquito’s cell. Understanding what happens at the cellular and molecular level will provide evidence on the sustainability of *Wolbachia* vector control.

## Introduction

*Aedes aegypti* is a target of vector control owing to its complete susceptibility to Dengue (DENV), Zika (ZIKV), and Chikungunya (CHIKV) viruses. Substantively, these arboviruses are detectable from mosquito populations worldwide resulting in geographical spread of human infections (Espinal et al., 2019). To curb increasing prevalence of arboviral diseases, vector control has been at the forefront with *Wolbachia* drawing considerable attention this decade (Indriani et al., 2020). The use of *Wolbachia* to control arboviral transmission to humans stems from its ability to persistently establish itself in a diverse vector species (Shaikevich et al., 2019). *Wolbachia pipentis* naturally resides in approximately 65% of all insect species and in arthropods, this bacterium is known to be both parasitic and mutualistic (Rousset et al., 1992; Weeks and Breeuwer, 2001; Hilgenboecker et al., 2008; Riparbelli et al., 2012; Beckmann et al., 2017; LePage et al., 2017). *Wolbachia* manipulates host reproduction through male killing (Riparbelli et al., 2012), feminization (Rousset et al., 1992), parthenogenesis (Weeks and Breeuwer, 2001), and more commonly, cytoplasmic incompatibility (CI) (Beckmann et al., 2017; LePage et al., 2017). CI is induced by the deubiquitinating enzyme (DUB) encoded by a two-gene operon *cidA-cidB*, which causes embryonic mortality when infected males mate with either uninfected females or those with a different *Wolbachia* strain (Beckmann et al., 2017; LePage et al., 2017). Such principle became the basis for *Wolbachia* release programs aiming for mosquito population suppression or replacement (Indriani et al., 2020). Other than reproductive manipulation, multiple evidences prove that *Wolbachia* significantly confers protection to mosquitoes against viruses yet little information is known on the cellular mechanisms underlying its antiviral impact. This pathogen blocking effect coupled with efficient spread have contributed to its potential as vector control agent (White et al., 2017; Shaikevich et al., 2019).

Previous studies have reported that in general, *Wolbachia* elicits a pathogen blocking phenotype in arthropods either by priming the host’s immunity and/or fighting for scarce host cellular resources (Kambris et al., 2010; Caragata et al., 2016; Angleró-Rodríguez et al., 2017; Pan et al., 2018). For instance, *Wolbachia* transinfection in *Ae. aegypti* leads to the activation of antiviral mechanisms along with an elevated production of antimicrobial peptides (Angleró-Rodríguez et al., 2017; Pan et al., 2018). Similarly, *Anopheles gambiae* transiently infected with the endosymbiont displays an upregulated expression of malaria-related immune genes and reduction in *Plasmodium* infection, confirming *Wolbachia*’s direct influence on host immunity (Kambris et al., 2010). Other studies have also shown that host cellular resources mainly cholesterol is an essential determinant of *Wolbachia*’s viral inhibition in *Drosophila* (Caragata et al., 2013) whereas gene network analysis involving the same insect species and other RNA viruses reveal strong interactions between metabolism pathways related to host nutrient production and viral replication (Lindsey et al., 2021).

Existing review articles on *Wolbachia*’s pathogen blocking effect present a broad discussion encompassing various arthropod species (Caragata et al., 2016; Kamtchum-Tatuene et al., 2017; Lindsey et al., 2018; Pimentel et al., 2021). On the contrary, this review focuses on *Ae. aegypti* alone for the following reasons: *Ae. aegypti* is the main target of *Wolbachia* release programs therefore focusing on mechanisms specifically linked to this mosquito will provide significant information on the sustainability of vector control strategy. Also, pathogen blocking mechanisms are not conserved among host species which gives no guarantee that a particular cellular mechanism involved in pathogen blocking applies to all. *Ae. aegypti* is also unique in a sense that this mosquito has long been regarded as a novel *Wolbachia* host until natural infection has been recently reported (Coon et al., 2016; Carvajal et al., 2019). This characteristic together with the use of multiple *Wolbachia* strains for transinfection add more complexity to arboviruses-*Wolbachia-Ae. aegypti* interactions. Other review papers have also presented cellular mechanisms that covered competition for cholesterol and lipids, cellular stress, and immunity (Kamtchum-Tatuene et al., 2017; Lindsey et al., 2018). We now expand the coverage of these cellular mechanisms by adding direct viral inhibition *via* host cytoskeleton (Lu et al., 2020), antagonistic lipid modulation (Koh et al., 2020), and cellular regeneration (Ford et al., 2019) based on recent reports. We also incorporate new information on how *Wolbachia*’s density may not be a contributing factor to immunity contrary to previous findings. Furthermore, we present the corresponding host gene and protein changes linked to each cellular mechanism.

This review aims to explore *Ae. aegypti*’s cellular mechanisms which *Wolbachia* affects to block arboviruses. We begin by illustrating how these medically important viruses change the gene/protein expression patterns of the host cell and exploit corresponding cellular mechanisms to strengthen viral infection. Subsequently, we will discuss how *Wolbachia* directly or antagonistically interferes with *Ae. aegypti*-arbovirus interactions through a) inhibition of viral entry and replication, b) reduction of specific nutrients required in arboviral infection, c) increase in Reactive Oxygen Species (ROS) production and immunity, d) cellular regeneration to enhance midgut barrier, and e) regulation of genes with various cellular functions (Hussain et al., 2011; Hussain et al., 2011; Ford et al., 2019; Koh et al., 2020; Lu et al., 2020). Finally, we summarize the effects on *Ae. aegypti* when infected with either virus or *Wolbachia* or both.

## Molecular Interactions Between Arboviruses and *Ae. aegypti*

*Ae. aegypti*’s intrinsic ability to act as a vector for disease is supported by its genome ingrained with genes that regulate cellular mechanisms for arboviral infection and defense. The latest annotated reference genome (AaegL5) of this mosquito portrays a wider coverage of gene families like chemosensory receptors, glutathione S-transferase, and C-type lectin with some unique quantitative trait loci (chromosome 2), all of which are paramount to viral susceptibility (Adelman and Myles, 2018; Matthews et al., 2018). DENV, ZIKV, and CHIKV, to some extent, impose differential transcriptomic changes and alter the function of translated proteins in *Ae. aegypti* (Mukherjee et al., 2019). Specifically, these arboviruses cause differential expression of transcripts under cytoskeletal, replication/transcription, immunity, ROS, and metabolism to control *Ae. aegypti’s* intracellular environment (Xi et al., 2008; Ramirez and Dimopoulos, 2010; Colpitts et al., 2011; Carvalho-Leandro et al., 2012; Sim et al., 2013; Angleró-Rodríguez et al., 2017; Mukherjee et al., 2019; Zhao et al., 2019) DENV, ZIKV, and CHIKV induce these changes in the mosquito in order to thrive without being pathogenic, allowing them to complete their life cycle (Mukherjee et al., 2019). In this section, we concentrate on the effects of arboviral infection on *Ae. aegypti*’s transcriptome, with only a brief discussion of proteomic and metabolomic expression patterns. These changes will be discussed together with the cellular mechanisms they affect to demonstrate host-virus interactions. Altogether, we explore the mechanisms on how arboviruses enforce viral infection and *Ae. aegypti’s* responses at the cellular level.

### Completion of Arboviral Life Cycle and Evasion of *Ae. aegypti’s* Midgut Barriers Strengthen Infection

Arboviral infection in *Ae. aegypti* begins with the ingestion of a viremic blood meal followed by an Extrinsic Incubation Period (EIP). EIP is a period of viral incubation within the host where virus enters the midgut, passes through the hemolymph to reach other organs and culminates in the salivary glands for subsequent infection (Chan and Johansson, 2012). Throughout the EIP, arbovirus tries to take over the host cell by using the cytoskeleton and overcoming the midgut barrier.

**Figure 1:**
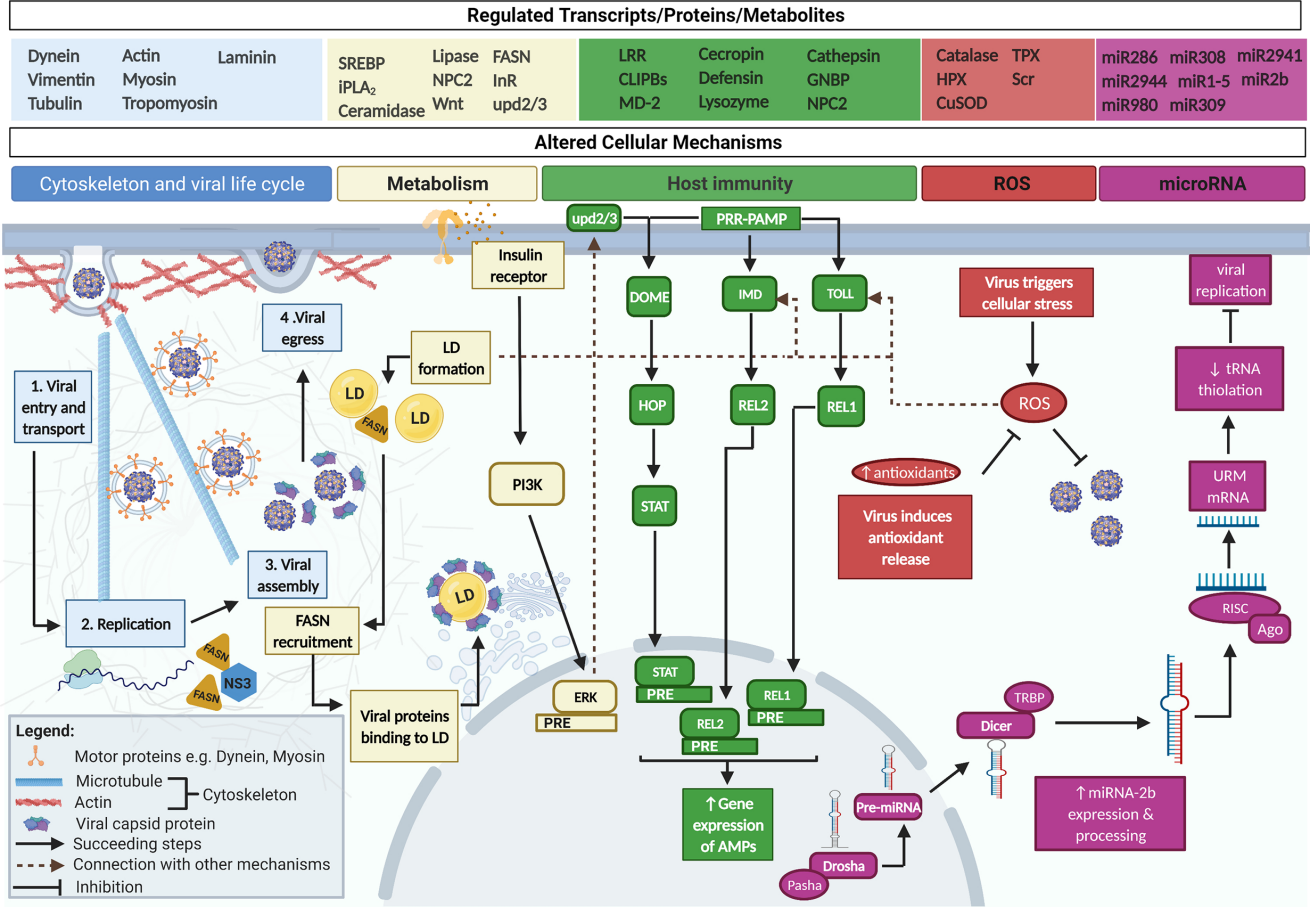
*Ae. aegypti*'s cellular mechanisms are affected by arboviral infection. Arboviral infections alter *Ae. aegypti*’s genes, proteins, and metabolites to control the host’s cellular mechanisms. First, arboviruses utilize host cytoskeletal proteins (e.g. dynein and myosin) to facilitate their intracellular transport. Second, the need for host cell nutrients allows the virus to alter host lipid/cholesterol and also triggers the formation of LD. LD can enhance host immunity *via* Toll/Imd, can aid viral replication through fatty acid synthase (FASN) recruitment by DENV NS3, and can be integrated into viral capsids. The activation of insulin receptor is also said to signal antiviral mechanisms i.e. ERK and JAK/STAT *via* upd2/3. Following activation of host immunity, antimicrobial peptides are produced. Third, arboviruses cause *Ae. aegypti* to produce elevated ROS. ROS can either directly harm invading arboviruses or activate the Toll immune mechanism. Arboviruses respond to the effects of ROS by upregulating antioxidant release which functions to lower down ROS in the cell. Fourth, miRNA-2b is initially processed inside the nucleus and brought out to the cytoplasm for further cleaving. A seed sequence produced from the RISC complex binds to URM, its target mRNA leading to the suppression of tRNA thiolation and inhibition of viral replication.

*Ae. aegypti’s* cytoskeleton, composed of a network of microtubules and actin filaments, is required for an infecting arbovirus to successfully traverse viral entry, replication, assembly, and egress **(** **Figure 1** **)** (Foo and Chee, 2015). During infection, arboviruses cause differential expression of *Ae. aegypti*’s cytoskeletal transcripts and proteins which are cellular components essential for viral life cycle. Genes like dynein, vimentin, tubulin, actin, myosin, tropomyosin, and laminin are highly expressed in DENV-infected *Ae. aegypti* (Bonizzoni et al., 2012; Sim et al., 2012) whereas CHIKV infection has shown marked cytoskeletal protein expression (Cui et al., 2020) as opposed to the uninfected. These gene/protein modifications within the host may represent the way arboviruses’ take advantage of the mosquito’s cytoskeleton by rearranging it into tracks to actively transport endosomes containing viral particles (Walsh and Naghavi, 2019). To facilitate transport at a later phase, direct interaction between arboviral proteins and host cytoskeletal motor proteins (e.g., dynein and myosin) occur (Paingankar et al., 2010; Mairiang et al., 2013; Foo and Chee, 2015; Walsh and Naghavi, 2019). In the case of DENV infection in *Ae. aegypti*, link between non-structural (NS) protein 5 and myosin has been reported (Mairiang et al., 2013). Similarly, other cytoskeletal structures like actin and tubulin is said to interact with DENV to facilitate infection *in vitro* (Paingankar et al., 2010). Protein interaction network prediction in *Ae. aegypti* also suggests that tubulin is highly associated with DENV infection in the mosquito host with roles in transport and assembly (Guo et al., 2010). In CHIKV infection, impaired viral delivery from the cell surface to the cytoplasm occurred when microtubule network is disrupted (Hoornweg et al., 2020). While ZIKV’s effect on *Ae. aegypti*’s cytoskeleton has not been fully explored, differential expression of cytoskeletal-related transcripts signifies that ZIKV likely uses the cytoskeleton for its entry, replication, assembly, and egress as other arboviruses (Etebari et al., 2017).

**Figure 2:**
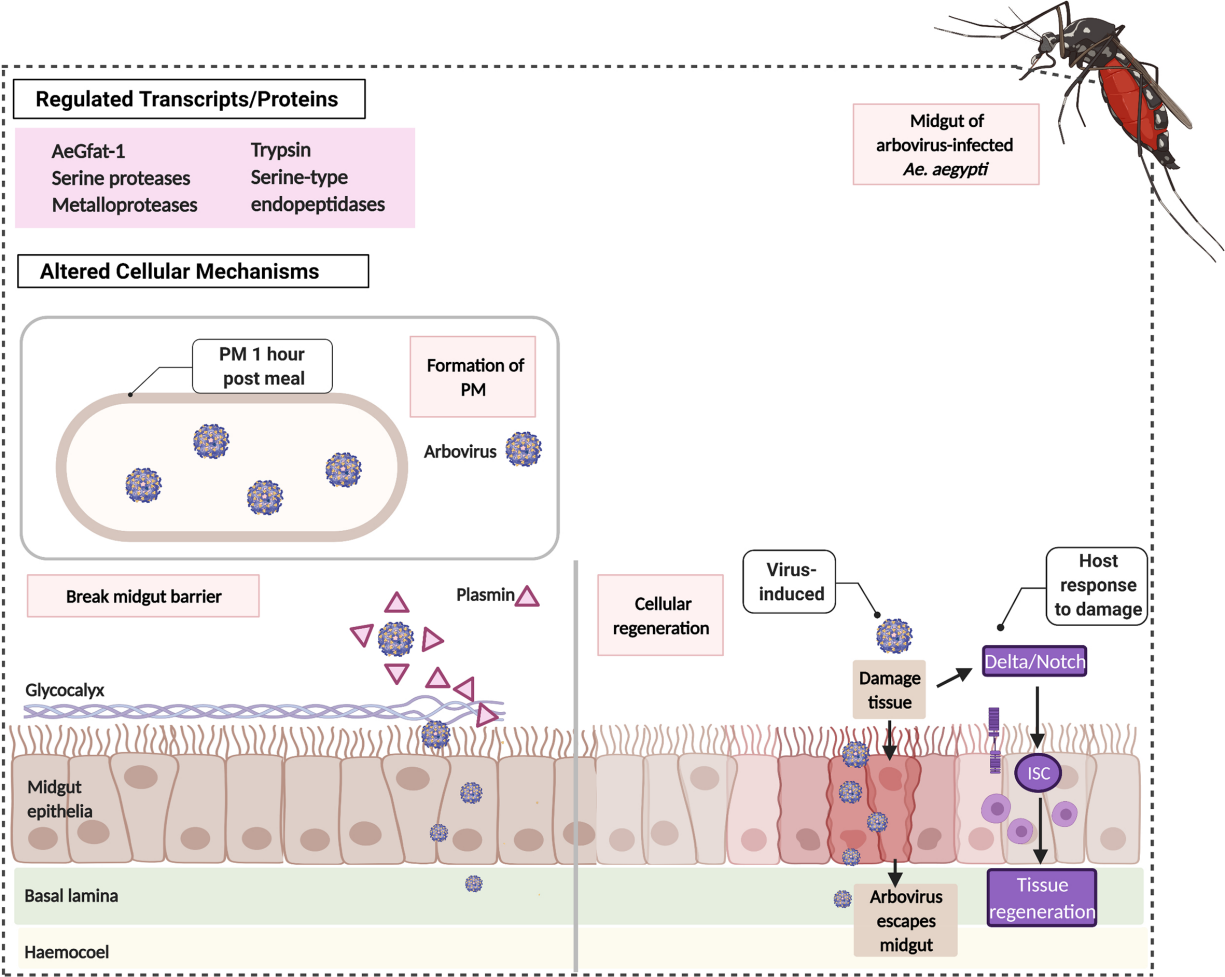
Arboviruses induce the formation of PM, cleaving of protective layer and Delta/Notch in *Ae. aegypti*’s midgut. An hour from the ingestion of virus-infected blood meal, *Ae. aegypti* forms a thick peritrophic membrane (PM) that encloses the virus separating it from the midgut epithelia. This PM prevents the virus from escaping the midgut and spreading the infection. Arboviruses may utilize proteolytic enzymes e.g. plasmin to break the glycocalyx, a protective layer at the surface of the midgut epithelia. Arboviruses may also directly damage the midgut tissue. *Ae. aegypti* can reverse this damage by activating the Delta/Notch resulting in the proliferation of Intestinal Stem Cells (ISC) for cellular regeneration.

Following a blood meal, *Ae. aegypti* transcribes the glucosamine fructose-6 phosphate aminotransferase (*AeGfAt-1*) gene involved in the formation of a chitinous sac called peritrophic membrane (PM) that encloses the ingested blood (**Figure 2**). PM formation happens in the midgut within 6 to 12 h post-feeding and is a physiological response for digestion (Kato et al., 2002; Suwanmanee et al., 2009). However, intake of blood with DENV forms a PM earlier, showing clear visibility just an hour after meal and is otherwise thicker (Suwanmanee et al., 2009). It has been speculated that PM could function as part of the Midgut Infection Barrier (MIB) by preventing the virus from penetrating the midgut epithelium and reaching other mosquito organs. This is consistent with the reports that demonstrate how PM hinders systemic infection in other vector mosquitoes (Rodgers et al., 2017). Aside from the upregulation of *AeGfAt-1*, DENV-, ZIKV-, and CHIKV-infected *Ae. aegypti* markedly express proteolytic transcripts (e.g. serine proteases, metalloproteases, trypsin, serine type endopeptidases) concurrent with specific abundance in protein composition of serine type proteases (Sim et al., 2013; Etebari et al., 2017; Whiten et al., 2018; Cui et al., 2020). These proteases can break down other proteins that strengthen the midgut barrier and can therefore be utilized by arboviruses to assert systemic infection. Recent evidence demonstrates that in *Ae. aegypti* mosquitoes, a protease called plasmin enhances DENV infection by breaking glycocalyx, a layer covering midgut epithelia cells whereas inhibition of plasmin’s activity resulted in low infection **(** **Figure 2** **)** (Ramesh et al., 2019).

Although arboviruses exploit different host cellular structures, viral replication and dissemination inside the host can be reversed by *Ae. aegypti*’s ability to regenerate the midgut epithelium (**Figure 2**). Taracena et al. describes how the exposure of the midgut epithelium to stress i.e. presence of arbovirus can result in the damage of the tissue. To counteract this, the host stimulates the proliferation of Intestinal Stem Cells (ISCs) responsible for cellular regeneration *via* a signaling pathway referred to as the Delta/Notch. Delta/Notch is able to increase DENV susceptibility of a refractory strain of *Ae. aegypti* when inhibited (Taracena et al., 2018). Hence, cellular regeneration *via* the Delta/Notch pathway is another important cellular response for arboviral blockade in *Ae. aegypti*.

### Metabolic Changes Facilitating Viral Replication and Inhibition

Arboviruses take over *Ae. aegypti*’s metabolism by disrupting lipid homeostasis intracellularly as demonstrated in the mosquito’s altered transcriptome and metabolome. *Ae. aegypti* infected with either of these arboviruses demonstrates high abundance of differentially expressed genes/proteins for lipid biosynthesis **(** **Figure 1** **)** (Tchankouo-Nguetcheu et al., 2010; Raquin et al., 2017; Royle et al., 2017; Shrinet et al., 2017; Chotiwan et al., 2018; Fukutani et al., 2018). DENV infection in particular upregulates sterol regulatory element-binding protein (*SREBP*), calcium independent phospholipase A2 (*iPLA_2_*), ceramidase, lipase, Niemann pick-type C2 protein (*NPC2*), and Wnt pathway regulator genes significantly relative to uninfected control (Raquin et al., 2017). Moreover, elevated glycerophospholipids, glycerolipids, sphingolipids, and fatty acids are found not just in DENV-infected *Ae. aegypti* but also those infected with ZIKV and CHIKV. All in all, these represent a significant increase in lipids during infection (Tchankouo-Nguetcheu et al., 2010; Royle et al., 2017; Chotiwan et al., 2018). The exact cellular pathway that links these genes and metabolites to virus infection remain undiscovered. *SREBP* is the only gene whose function has been directly attributed to promote DENV infection whereas its knockdown decreases the viral load significantly (Raquin et al., 2017).

Further reports have associated high lipids with the viral replication and formation of lipid rich structures called lipid droplets (LD; **Figure 1** **)** (Heaton et al., 2010; Barletta et al., 2016). Initially, LD is regarded as a reservoir for cholesterol but has later been discovered to have a dynamic role. LD formation is accompanied by an increase in fatty acid synthase enzyme that catalyzes LD metabolism during DENV and CHIKV infection (Heaton et al., 2010; Tchankouo-Nguetcheu et al., 2010). Fatty acid synthase is said to be recruited by DENV NS3 at the site of replication in the absence of other NS proteins. Alongside its role in viral replication, LD is incorporated in the viral capsid during assembly. LD has also been linked to antiviral immunity in *Ae. aegypti* given that its accumulation strengthens the host’s immunity by activating Toll and Immune deficiency (Imd) cellular mechanisms (Barletta et al., 2016).

Interestingly, recent finding suggests that insulin and its receptor strengthen immunity against DENV and ZIKV infection in *Ae. aegypti* (Ahlers et al., 2019) by activating Janus kinase/signal transducers and activators of transcription (JAK/STAT). When mosquito cells are treated with insulin, the insulin receptor (InR) activates a cellular mechanism called the phosphatidylinositol 3’-kinase (PI3K) signaling pathway that send signals to the extracellular signal-regulated kinases (ERK). Once ERK is activated, it is said to prompt the release of molecules under the unpaired (upd2/3) family. Upd2/3 then engages the Domeless receptor (Dome), that sends signals to downstream proteins under the JAK/STAT leading to arboviral inhibition (Ahlers et al., 2019).

### The Tug of War Between Host’s Immune System and Viral Infection

The involvement of the host’s immunity as a key player in regulating arboviral infection has been corroborated in several studies (Xi et al., 2008; Colpitts et al., 2011; Carvalho-Leandro et al., 2012; Sim et al., 2013; Angleró-Rodríguez et al., 2017; Caicedo et al., 2019; Mukherjee et al., 2019; Zhao et al., 2019). Arboviruses cause *Ae. aegypti* to actively respond to infection by switching immune-related genes on/off and utilizing the same genes to dictate the extent of viral susceptibility. This manifests at the transcriptomic level where high expression of leucine rich repeat (LRR)–containing proteins, Clip-domain serine proteases (CLIPBs), and Myeloid differentiation 2–related lipid recognition protein (MD-2) receptors are noted (**Figure 1**) (Xi et al., 2008; Angleró-Rodríguez et al., 2017; Caicedo et al., 2019; Zhao et al., 2019). LRR, CLIPBs, and MD-2 initiates *Ae. aegypti*’s immunity by functioning as Pattern Recognition Receptors (PRRs). PRRs recognize viral particles as Pathogen-Associated Molecular Patterns (PAMPs) at the surface of the host cell (Xi et al., 2008; Mukherjee et al., 2019). Consequently, the PRR-PAMPs binding causes Upd ligands to associate with Dome stimulating JAK tyrosine kinase Hopscotch (Hop) to phosphorylate. In turn, STAT proteins are activated and translocated to the nucleus where it is able to bind to the palindromic response element (PRE) to induce gene expression. The same signaling cascade applies to the Imd and Toll pathways only that different ligands and receptors are activated. Downstream of these pathways are proteins referred to as Relish (REL2, REL1) that binds to the PRE in the nucleus and triggers the expression anti-microbial peptides (AMPs) i.e. cecropin, defensin, lysozyme, and cathepsin (Hoffmann, 2003; Jang et al., 2006; Mukherjee et al., 2019). AMPs can directly kill microbes or further enhance the immunity. In both midgut and carcass tissue of DENV-, ZIKV-, and CHIKV-infected *Ae. aegypti*, AMP transcripts are highly evident (Xi et al., 2008; Sim et al., 2013; Angleró-Rodríguez et al., 2017). More so, silencing Cactus and Caspar that are inhibitory to Toll and Imd resulted in enhanced antimicrobial peptide gene expression (Xi et al., 2008). Indeed, these studies provide the basis for *Ae. aegypti*’s natural ability to resist viral infection. Contrary to this, viruses can increase host arboviral susceptibility by downregulating the same AMP genes as seen in transcriptomic and *in vitro* studies of DENV-, ZIKV-, and CHIKV-infected mosquitoes (Sim and Dimopoulos, 2010; Colpitts et al., 2011; Carvalho-Leandro et al., 2012). Some immune-related genes have also been directly linked to susceptibility. In a recent study by *Caicedo et al.*, genes such as Gram-negative binding protein – GNBP *(AAEL009176)*, NPC2 *(AAEL015136)*, Keratinocyte lectin *(AAEL009842)* and Cathepsin-b *(AAEL007585)* when inhibited in a susceptible strain of *Ae. aegypti* led to a significant reduction in DENV dissemination (Caicedo et al., 2019). This proves the importance of these genes and their corresponding functions in DENV infection.

### Inhibition of Reactive Oxygen Species Promotes Arboviral Infection

Arboviral infection results in production of ROS as part of the physiological response of *Ae. aegypti* to cellular stress (**Figure 1**). Excess ROS production act as secondary messengers that signals the innate immune response and can directly damage invading microbes (Tchankouo-Nguetcheu et al., 2010; Bottino-Rojas et al., 2018). To ensure infection, these arboviruses can neutralize the upregulated activity of ROS antiviral defense by modifying the gene expression profile of *Ae. aegypti*. Transcripts classified as antioxidants are markedly increased in arbovirus susceptible mosquito host. These antioxidants scavenge the ROS to decrease its harmful effects (Oliveira et al., 2017; Shrinet et al., 2018; Wang et al., 2019). During DENV infection, the antioxidant catalase functions to balance ROS and increase DENV concentration in mosquito’s midgut epithelia (Oliveira et al., 2017). Antioxidant hemopexin (HPX) exerts the same effect and enhances not just DENV infection but also ZIKV in *Ae. aegytpi* (Wang et al., 2019). Concomitantly, antioxidant transcripts like superoxide dismutase (CuSOD), thioredoxin peroxidase (TPX), and scavenger reporter (Scr) in *Ae. aegypti* infected with individual DENV/CHIKV as well as co-infection have demonstrated significant abundance (Shrinet et al., 2018). Downregulating the expression of antioxidants results in the reduction of DENV, ZIKV, and CHIKV titer (Oliveira et al., 2017; Wang et al., 2019). Similar to the manner by which virus directly exploits host immunity to their advantage, arboviruses must also ensure that oxidative stress represented by an increase in ROS is circumvented by adequate antioxidant release.

### Micro-RNAs Regulate *Ae. aegypti* Genes That Affect Viral Infection

Arboviruses utilize *Ae. aegypti’s* micro-RNAs (miRNAs) for infection albeit the host can also use these for viral inhibition. miRNA is a type of non-coding RNA (ncRNA) that bind to DNA/RNA to either enhance or suppress the function of genes under various functional categories (Campbell et al., 2014). Initially, primary miRNAs (pri-miRNAs) undergo cleaving by Drosha and Pasha within the nucleus and exported to the cytoplasm as precursor miRNAs (pre-miRNAs) (**Figure 1**). Subsequently, pre-miRNAs go through additional modifications *via* Dicer to generate about ~22 nt base paired strands known as mature miRNA. This miRNA is loaded onto the RISC complex composed of Argonaute (Ago) proteins involved in the selection of one strand (aka guide strand). This guide strand constitutes two to six nucleotides referred to as the seed sequence that directly binds to the target mRNA for regulation. In general, arboviral infection give rise to a significant decline in *Ae. aegypti* global miRNA expression (Dubey et al., 2017; Saldaña et al., 2017; Dubey et al., 2019). Saldaña et al. reported 17 miRNAs that are significantly regulated in three time points during ZIKV infection. Maximum negative fold change has been observed in *aae-miR-286a, aae-miR-2944b-3p*, *aae-miR-980-3p* at 2 days post infection (dpi) and *aae-miR-308-3p* (7 dpi) whilst *aae-miR-2940-3p* and *aae-miR-1-5p* are enriched at 14 dpi. In addition, the abundance of some of the miRNAs particularly *aae-miR-309-a* and *aae-miR-2941* are altered (Saldaña et al., 2017). A comparable observation has been reported in DENV2-infected *Ae. aegypti* (Campbell et al., 2014) but further studies investigating their target genes and effects are warranted. To illustrate the role of miRNA during virus infection, miRNAs and its potential targets in *Ae. aegypti* infected with CHIKV have been analyzed. *miR-2b* is one of the most significantly upregulated miRNA said to regulate ubiquitin-related modifier (URM), a cellular factor that promotes CHIKV replication (Dubey et al., 2017). This miRNA binds to the URM gene thereby exerting a suppressive effect on tRNA thiolation, a process required for gene translation. By inhibiting tRNA thiolation, CHIKV viral load is reduced (Dubey et al., 2017). Additionally, *miR-2944b-5p* has been said to enhance vps-13 expression which in turn stabilizes CHIKV in *Ae. aegypti* cells by maintaining mitochondrial stability (Dubey et al., 2019).

## *Wolbachia* as an Arboviral Inhibitor

Mass release programs have been implemented to deploy *Wolbachia* in different communities that so far led to a reduction in dengue cases (O’Neill et al., 2019). Indeed, *Wolbachia pipentis* from other species when transinfected into *Ae. aegypti* do not only induce CI to suppress the mosquito population but also inhibits arboviruses (Lindsey et al., 2018). However, there is growing evidence that different *Wolbachia* strains carry varying blocking effects in *Ae. aegypti* with some strains failing to reduce viral replication and transmission despite being present at high density **(** **Table 1** **)** (Flores et al., 2020; Fraser et al., 2020). Under *Wolbachia* supergroup A, *w*AlbA is able to reduce ZIKV infection rates in orally infected *Ae. aegypti* but not in mosquitoes with intrathoracic ZIKV infection and oral/intrathoracic DENV infection (Chouin‐Carneiro et al., 2020). Meanwhile, *Wolbachia* strains *w*MelPop and *w*MelPop-CLA confers immune protection against DENV and CHIKV whereas the former strain together with *w*Mel alters lipid/cholesterol content for DENV and ZIKV blocking (Moreira et al., 2009; Geoghegan et al., 2017; Asad et al., 2018; Fraser et al., 2020; Koh et al., 2020; Manokaran et al., 2020). Furthermore, *w*Mel exhibits an anti-ZIKV effect that regulates the insulin receptor potentially suggesting another metabolic regulation (Haqshenas et al., 2019) as well as anti-DENV cellular regeneration (Ford et al., 2020). Meanwhile, *Wolbachia* strain *w*Pip from the supergroup B has been tested on *Ae. aegypti* where it did not enhance the mosquito’s innate immunity against DENV (Fraser et al., 2020) while in other studies *w*AlbB directly intervenes with viral infection and increases ROS for immune activation (Pan et al., 2012; Lu et al., 2020). These studies may indicate that aside from *Wolbachia* strain, the variability in arboviral inhibition may be attributed to the mechanism/s and the extent to which these mechanisms are regulated by each strain inside the host.

**Table 1:** Mechanistic effects of multiple *Wolbachia* strains in arbovirus-infected *Ae. aegypti*.

Wolbachia strain	Natural host	Mechanistic effect on *Ae. aegypti*	Virus	References
Supergroup A				
*w*AlbA	*Ae. albopictus*	Did not reduce oral/intrathoracic viral infection	DENV (oral and intrathoracic), ZIKV (intrathoracic)	Chouin-Carneiro et al., 2020 ^b^
		Reduced oral infection	ZIKV only	
*w*MelPop	*D. melanogaster*	Increase in cholesterol cellular content	DENV	Geoghegan et al., 2017 ^a^
		Immunity	DENV	Fraser et al., 2020 ^b^
*w*MelPop-CLA	*D. melanogaster*	Immunity	DENV, CHIKV	Moreira et al., 2009 ^b^ Asad et al., 2018 ^a,b^
*w*Mel	*D. melanogaster*	Increase in cholesterol cellular content	DENV	Geoghegan et al., 2017 ^a,b^
		Decrease selected lipids necessary for viral infection	DENV, ZIKV	Koh et al., 2020 ^b^ Manokaran et al., 2020 ^a^
		Reduced activity of insulin receptor	ZIKV	Haqshenas et al., 2019^a^^,^^b^
		Little expression of defensin and cecropin. Not comparable with *w*MelPop	DENV	Fraser et al., 2020^b^
		Cellular regeneration	DENV	Ford et al., 2020 ^b^

Supergroup B				
*w*AlbB	*Ae. albopictus*	Direct inhibition of viral binding and entry	DENV, ZIKV	Lu et al., 2020 ^a^
		ROS-mediated toll activation	DENV	Pan et al., 2012 ^b^
*w*Pip	*Cx. quinquefasciatus*	Did not confer protective immunity	DENV	Fraser et al., 2020 ^b^

a in vitro.

b in vivo.

Multiple Wolbachia strains under supergroups A and B have been found to induce varying pathogen blocking effects in Ae. aegypti. These strains are tested either in vitro, in vivo, or both.

The previous sections explained how arboviruses exploit *Ae. aegypti* cellular machinery by utilizing the mosquito’s cytoskeletal elements for viral infection and proteolytic enzymes to break the midgut barrier. In addition, arboviral infections take advantage of the host nutrients for their propagation. Counteracting ROS through antioxidant release and miRNA generation are also means for creating a conducive intracellular environment for these arboviruses to thrive. In the following sections, we present how *Wolbachia* adds another layer to *Ae. aegypti*-arbovirus interaction by interfering with the same molecular mechanisms and inducing cellular perturbations that are detrimental to the pathogen.

### *Wolbachia* Uses *Ae. aegypti’s* Cytoskeleton to Inhibit Viral Binding and Entry

**Figure 3:**
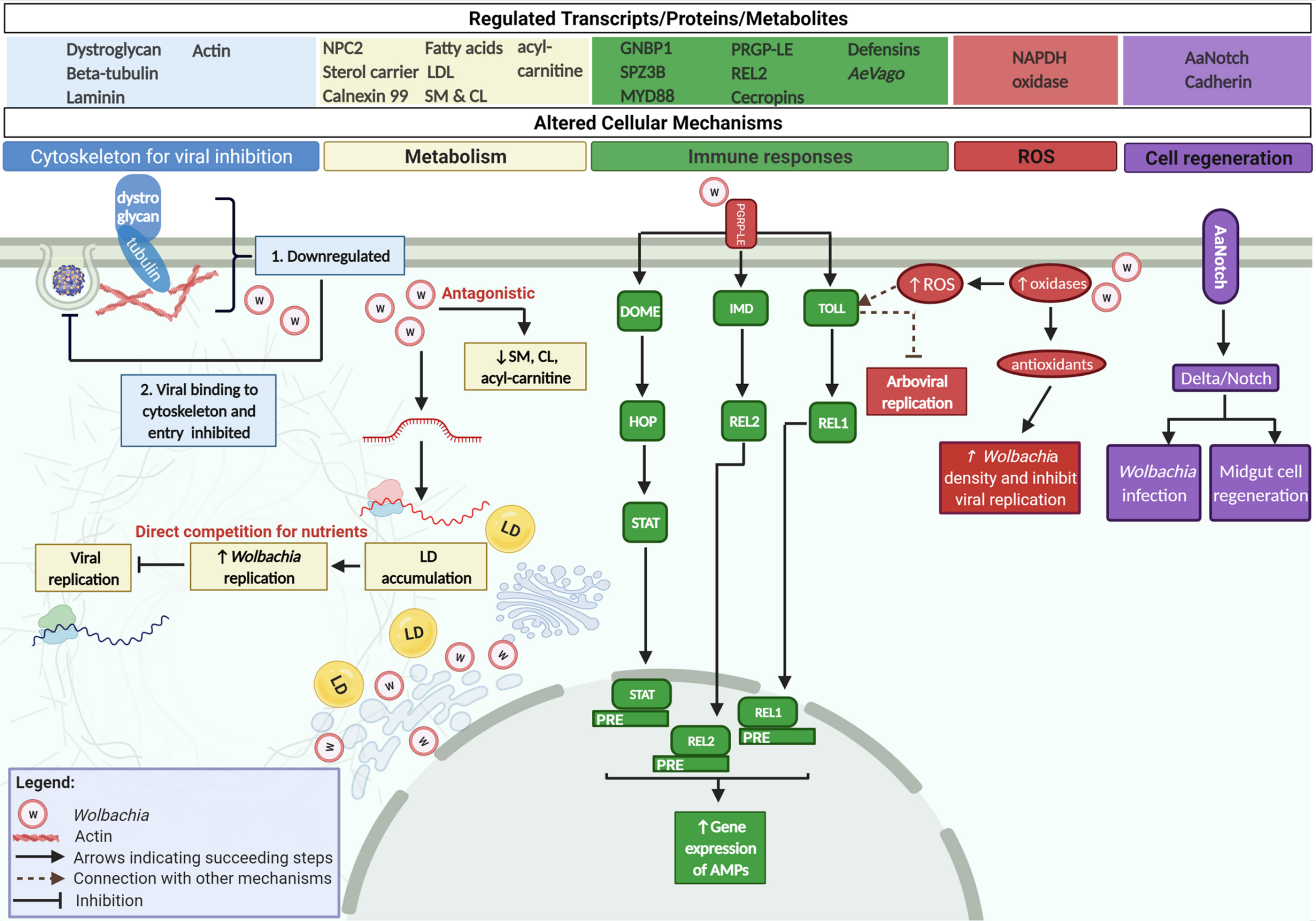
*Wolbachia* interferes with Arboviral life cycle by interfering with host factors and strengthening host antiviral mechanisms. *Wolbachia* blocks DENV, ZIKV, and CHIKV by interfering with the same cellular mechanisms arboviruses control in *Ae. aegypti.* First, *Wolbachia* downregulates cytoskeletal structures that directly bind to arboval proteins for host cell infection. Next, *Wolbachia* competes for host’s lipid/cholesterol for its replication making this nutrient insufficient for the virus. *Wolbachia* can also deplete lipids namely sphingomyelins (SM), cardiolipins (CL), and acyl-carnitine that are essential for viruses. *Wolbachia* also strengthens the host’s immunity by activating Toll, IMD, and JAK/STAT *via* PGRP-LE. The endosymbiont further induces ROS release in the presence of an arbovirus by increasing oxidases in the cell. This inhibits viral replication and in turn, stimulates the Toll pathway. *Wolbachia* then counteracts the release of antioxidants to sustain its infection within the host. Lastly, *Wolbachia* upregulates receptor gene, *AaNotch* in *Ae. aegypti*. This receptor gene signals the Delta/Notch pathway. Consequently, the adhesion molecule Cadherin is increased strengthening the host’s reproductive ability and ensures maintenance of *Wolbachia.* Delta/Notch also promotes the regeneration of the midgut epithelia to prevent systemic viral infection.

Studies have attributed *Wolbachia’s* pathogen blocking effect on its ability to decrease viral load. The mechanism by which this effect is accomplished, as well as the point in the virus’ life cycle at which such interference occurs, are previously unknown. The present study reveals that transinfected *w*AlbB strain in *Ae. aegypti* (Aag2) cell line infected with either DENV or ZIKV caused the downregulation of cytoskeletal membrane proteins, dystroglycan and beta-tubulin (**Figure 3**) (Lu et al., 2020). Concurrently, viral binding assays display a significant reduction in viral RNA copy number as early as 2 h post infection suggestive of an early viral interference in DENV and ZIKV binding as well as entry. Further validation done by silencing both cytoskeletal membrane proteins inhibited DENV binding to Aag2 cells (Lu et al., 2020). This is the first demonstration to confirm the direct involvement of *Wolbachia* in arbovirus binding and entry by taking advantage of the same host cytoskeletal proteins DENV and ZIKV utilize. Although there is a lack of data that demonstrates the same mechanism in CHIKV, this arbovirus necessitates an intact microtubule network consisting of alpha-tubulin (same family as beta-tubulin) for efficient viral entry and genome delivery to replication sites (Hoornweg et al., 2020).

In other host species that harbor *Wolbachia* infection, cytoskeletal proteins are vital for maintaining the endosymbiont’s density (Baldridge et al., 2014; Sheehan et al., 2016). Native *Wolbachia* in *Drosophila* expresses a type IV secretion system (T4SS) that releases an effector molecule called *Wolbachia* actin-localizing effector 1 protein (WalE1) (Sheehan et al., 2016). WalE1 directly binds to actin aiding in *Wolbachia*’s localization within the host (Sheehan et al., 2016). This is consistent with the report on significant reduction of the endosymbiont’s density coupled with inefficient maternal transmission when mutations in the actin gene are present (Newton et al., 2015). In like manner, *Ae. albopictus* expresses T4SS and a corresponding WalE1 homolog which may suggest contribution to the maintenance of *Wolbachia* infection in this species (Baldridge et al., 2014).

Based on the evidence, *Wolbachia* interacts with the host cytoskeleton in two ways. First, its ability to secrete effector molecules that bind to cytoskeletal structures ensures that *Wolbachia* resides in the host cell at an optimal density and guarantees transmission to other hosts. Second, *Wolbachia* regulates the expression of cytoskeletal proteins like dystroglycan and tubulin, required for arboviral infection (Lu et al., 2020). It is remarkable how arboviruses and *Wolbachia* have contrasting effects on the host cytoskeleton. While the former upregulates cytoskeletal structures for virus entry, replication, assemble, and egress, the latter downregulates the same structures to block arboviral binding/entry (Sim et al., 2012; Sim et al., 2013; Lu et al., 2020).

### Lipid Perturbations Caused by *Wolbachia* Are Antagonistic to Arboviruses

*Wolbachia* localizes within golgi-related vesicles in close proximity to the endoplasmic reticulum, the site of cell membrane biogenesis (Cho et al., 2011). Provided that membrane biogenesis relies on host lipids, this position allows *Wolbachia* to acquire nutrients for itself (Fattouh et al., 2019). Previous reports show that *Ae. albopictus* cells infected with *w*Mel resulted in decreased sphingolipids, diacylglycerol, and phosphatidylcholine (Molloy et al., 2016). These host lipids constitute structures known as lipid rafts used by the bacteria to enter the cell and activate mechanisms required for bacterial invasion (Lafont and Goot, 2005). Likewise, *Ae. aegypti* cells infected with *w*MelPop or *w*Mel experienced 15% to 25% reduction in total cholesterol signifying *Wolbachia’s* dependence on host cellular lipids (Caragata et al., 2014). The presence of either *Wolbachia* strain and DENV in *Ae. aegypti* perturbs cholesterol levels where an increase in stored cholesterol and localized accumulation of LD has been observed (**Figure 3**) (Geoghegan et al., 2017). This is represented by an upregulation of Niemann-Pick type C2 (*NPC2*), sterol carrier protein 2, calnexin 99 and a simultaneous downregulation of fatty acid synthase and LDL receptor proteins corresponding to an impaired intracellular cholesterol transport (Geoghegan et al., 2017). More so, treatment of *Ae. aegypti* infected with *Wolbachia* using a substance for stabilizing cholesterol resulted in the restoration of DENV replication (Geoghegan et al., 2017). Cholesterol supplementation that rescued viral replication has first been noted in other arthropod species such as *Drosophila* and *Ae. albopictus* (Caragata et al., 2013; Schultz et al., 2017). Several studies then pointed out that these metabolic changes induced by *Wolbachia* could be responsible for the pathogen-blocking mechanism in transinfected *Ae. aegypti*. Significant increase in the host’s cholesterol content takes place due to *Wolbachia’s* sequestration of cholesterol and lipid droplets, the same nutrients crucial for DENV, ZIKV, and CHIKV replication (Geoghegan et al., 2017; Cui et al., 2020; Leier et al., 2020). *Wolbachia* and arboviruses’ need for the same resources become the theoretical basis for competition between the two microbes with *Wolbachia* surpassing viral infection for its self-preservation (Caragata et al., 2013; Geoghegan et al., 2017; Schultz et al., 2017; Leier et al., 2020). Conversely, recent studies propose that instead of competition for lipids, antagonistic lipid modulation occurs (Koh et al., 2020; Manokaran et al., 2020). In the study by Koh et al., mono-infection of DENV results in lipid abundance whereas *w*Mel causes mild depletion. However, co-infection of DENV and *w*Mel in *Ae. aegypti* displays a lipid profile that resembles DENV induced perturbations signifying arbovirus control while certain lipids have been reported to be indirectly antagonistic (Koh et al., 2020). Take for example sphingomyelins (SM) and cardiolipins (CL) which were enriched in DENV3-infected *Ae. aegypti* but depleted in the presence of the same virus and the endosymbiont. CL knockdown also decreased DENV load regardless of *w*Mel’s presence but replication of *w*Mel is impaired only in the absence of DENV (Koh et al., 2020). This antagonistic interaction also applies in another study where another class of lipids called acyl-carnitine is elevated during DENV and ZIKV infection but significantly diminished in *w*Mel-infected Aag2 cells (Manokaran et al., 2020). Reduction in acyl-carnitine enhanced *w*Mel density yet addition of the said lipid in Aag2 containing *w*Mel boosts DENV and ZIKV infection (Manokaran et al., 2020).

*Wolbachia* is similar with arboviruses in a sense that both are unable to synthesize lipids making them dependent on the host cell. While this competition means that one must utilize host nutrients at the expense of the other, recent findings show us that co-infection of these microbes within *Ae. aegypti* may also entail an antagonistic interaction. Perhaps the virus tries to establish an intracellular environment high in lipids for its replication which *Wolbachia* hampers.

Meanwhile, other factors that play a role in lipid homeostasis is insulin which promotes fat storage in cells. Latest study reveals that DENV and ZIKV suppression is mediated by the downregulation of insulin receptor in *w*Mel-transinfected *Ae. aegytpi* (Haqshenas et al., 2019). In the previous section, insulin has been linked to the activation of host immune system. The underlying interaction between *Wolbachia* and arboviruses in the context of this mechanism warrants further investigation.

### *Wolbachia* Induces Variable Immunity in *Ae. aegypti* and the Role of Reactive Oxygen Species

Existing view posits that transinfected *Wolbachia* infection in *Ae. aegypti* primes the mosquito’s immune system prior arboviral infection (Pan et al., 2018) yet the extent of immune activation differs among *Wolbachia* strains. Similar with the host’s response to virus, *w*AlbB*-*transinfected *Ae. aegypti* upregulates genes under the Toll (GNBP1, SPZ3B, MYD88) and Imd (PRGP-LE, REL2) pathways leading to antimicrobial peptide (e.g. cecropins, defensins) release during arboviral infection (**Figure 3**) (Xi et al., 2008; Angleró-Rodríguez et al., 2017; Pan et al., 2018; Zhao et al., 2019). Furthermore, innate immunity can be activated by ROS indicating cross-talk between immune and redox mechanisms (Pan et al., 2012). With DENV infection, *w*AlbB stimulates the production of NAPDH oxidases which are key producers of ROS. ROS then activates the Toll pathway to reduce DENV load. These oxidases, when silenced, deactivate the host’s immunity leading to an increased DENV titer (Pan et al., 2012). Simultaneously, Toll controls antioxidant expression that helps *w*AlbB maintain its infection in *Ae. aegypti* (Pan et al., 2012). It is interesting to note that ROS-dependent immune pathway activation does not occur in *Wolbachia’s* natural host, *Ae. albopictus* (Molloy and Sinkins, 2015). Adult mosquito lines carrying transinfected *w*Alb and *w*Mel that varies in density show neither an upregulation in ROS-related genes nor innate immune activity (Molloy and Sinkins, 2015). In the same study, this effect has been observed *in vitro* using *w*MelPop, *w*Mel, and *w*AlbB transinfection suggesting that ROS-dependent immune activation could be unique to *Ae. aegypti* (Molloy and Sinkins, 2015).

Meanwhile, *w*Melpop-CLA blocked DENV and CHIKV *via* JAK/STAT in *Ae. aegypti* (Moreira et al., 2009; Asad et al., 2018). *w*Melpop-CLA inhibits DENV through an upregulation of a Vago protein homolog (*AeVago*) that activates JAK/STAT (Asad et al., 2018). In CHIKV, this strain has been observed to cause an upregulation of AMPs but displayed unparalleled results in two independent experiments in terms of transcriptional modulation of immune-related genes (Moreira et al., 2009). All in all, multiple strains of *Wolbachia* demonstrate arboviral blocking that is partly mediated by the host’s immunity yet there remains an underlying issue on the magnitude of this effect. And whether this immunity merely depends on the strain or density is still debatable.

A recent study that compared multiple *Wolbachia* strains in a consistent *Ae. aegypti* line provided deeper understanding of viral blocking based on the endosymbiont’s strain and density (Fraser et al., 2020). Here, transinfected *w*Pip at high density in *Ae. aegypti*, failed to block DENV replication and dissemination (Fraser et al., 2020). Notably, *w*Pip together with DENV-inhibitory *w*AlbB and *w*Mel only induce very small to no increase in expression of genes under the Toll pathway. Marked upregulation in immune-related genes has only been observed in *w*Melpop (Fraser et al., 2020). Contrary to other hypotheses, this study demonstrates that there is no association between *Wolbachia* strain/density and the extent of immune activation to block DENV. It is also interesting to highlight how *w*Mel can effectively restrict DENV infection yet with only a small upregulation of *Ae. aegypti’s* immunity (Fraser et al., 2020). *w*Mel is the strain that is predominantly used in mass release programs and is said to work against arboviruses by manipulating the host’s lipid/cholesterol (Geoghegan et al., 2017; Haqshenas et al., 2019; Koh et al., 2020; Manokaran et al., 2020). This then opens the possibility that specific *Wolbachia* strains induce their antiviral effect using different, not necessarily all of the host cellular mechanisms.

*Wolbachia* strains when traced back to their natural hosts may suggest potential pathogen blocking activity but it does not automatically lead to the same effect when transinfected in *Ae. aegypti* and in the case that it does, the immune pathways *Wolbachia* strains activate may not be conserved. Other than the strain or density per se, the regulation of innate immunity in *Ae. aegypti* seems more complex and influenced by intracellular responses that have yet to be elucidated.

### Cellular Regeneration—a feature of *Ae. aegypti* With High *Wolbachia-*Mediated Viral Blocking

New inferences on the molecular mechanisms of *Wolbachia*-mediated viral blocking in *Ae. aegypti* involves the Notch signaling pathway and cell-cell adhesion (**Figure 3**). Generally, Notch is a conserved mechanism that enhances the host’s fitness (Kopan and Ilagan, 2009; Chang et al., 2018). In fact, *Ae. aegypti* carries a Notch receptor gene (*AaNotch)* involve in maintaining micropyle pores and fecundity, all of which contribute to overall reproductive ability (Chang et al., 2018). Notably, this high fitness advantage in *Ae. aegypti* has been recently associated with stronger *Wolbachia*-mediated viral blocking (Ford et al., 2019; Ford et al., 2020). In the study by Ford et al., wMel-infected *Ae. aegypti* artificially selected for high *w*Mel-mediated viral inhibition displays a gene profile in favor of Notch activation which significantly differs from mosquitos classified with low *w*Mel-mediated DENV blocking. Notch activation coupled with high viral blocking has been linked to elevated cadherin expression (Ford et al., 2019; Ford et al., 2020). Although the mechanistic relationship between Notch and cadherin in *Ae. aegypti* has not been explicitly defined, a study in *Drosophila* shows how these two combine as a single complex to form cell-cell junctions implicated in Delta/Notch activation. Moreover, depletion of cadherin results in a downregulated activity of the said mechanism (Sasaki et al., 2007).

Consistently, studies on host-virus interactions also provided more context to this potential mechanism by proving that Notch has a regulatory role on midgut cell proliferation in *Ae. aegypti* during DENV infection. This proliferative response is referred to as endoreplication, a process that promotes tissue regeneration by producing cells with excess copies of genomic DNA (Serrato-Salas et al., 2018; Taracena et al., 2018). In a susceptible *Ae. aegypti* strain, delayed cellular regeneration has been observed upon DENV infection whereas induction of the midgut cell proliferation made this mosquito more resistant to the virus. Alternatively, *Ae. aegypti* refractory strain demonstrates higher susceptibility to DENV upon inhibition of Notch (Taracena et al., 2018). These findings therefore suggest that in *Ae. aegypti* the Notch specifically regulates *Wolbachia’s* viral blocking activity by increasing both host fitness and midgut cellular regeneration.

### miRNAs Solely Expressed During *Wolbachia* Infection Blocks Arboviruses

miRNA functions as a post-transcriptional regulator of gene expression and has a wide-ranged effect given its control over multiple genes. Some miRNAs that usually exist within the host are dysregulated while others become exclusively expressed in the presence of microbes. Successively, microbes can drive these miRNAs to alter the mosquito host’s responses as they persist inside the cell (Feng et al., 2018) (**Table 2**).

**Table 2:** miRNAs induced by *Wolbachia* in *Ae. aegypti*.

miRNAs	Target genes	Activity on target gene	Proposed effect on arbovirus-infected *Ae. aegypti*
miR-2940	m41 ftsh, AaArgM3	enhance	*w*Melpop-CLA** density
	AaDnmt2	suppress	*w*Melpop-CLA** density
aae-miR-12	MCT1	suppress	downregulates autophagy controlled by the virus
	MCM6	suppress	*w*Melpop-CLA density
aae-miR-981	Importin β-4	suppress	blocks AGO1 translocation

WsRNA-46	dynein	enhance	*w*Melpop** replication and migration
			mRNA localization
			lipid droplet movement

The presence of Wolbachia in Ae. aegypti can trigger the release of miRNAs derived from the host (miR-2940, aae-mir-12, aae-miR-981) or the endosymbiont (WsRNA-46). These miRNAs can regulate different host cellular mechanisms to maintain Wolbachia’s density, facilitate transport, and strengthen host antiviral responses.

Studies have substantiated the impact of *Wolbachia* on *Ae. aegypti*’s miRNA profile (Hussain et al., 2011; Mayoral et al., 2014). *w*Melpop-CLA induces differential expression of miRNAs with exclusive induction of *miR-2940* and *miR-309a-2* in *Wolbachia*-positive mosquitoes (Hussain et al., 2011). Between the two, *miR-2940* is well-known to target genes that regulate *Wolbachia* density (Hussain et al., 2011; Zhang et al., 2013; Zhang et al., 2014). In particular, this miRNA increases and stabilizes the expression of metalloprotease m41 ftsh gene and arginine methyl transferase 3 *(AaArgM3)* further enhancing *w*Melpop-CLA replication in both *Ae. aegypti* cells and mosquitoes. Inhibiting miR-2940 only led to a significant reduction of the target genes and endosymbiont (Hussain et al., 2011; Zhang et al., 2014). The specific role of this *miR-2940* in arbovirus infection remains undiscovered although this miRNA is downregulated in mosquito cells to limit West Nile virus replication (Slonchak et al., 2014). Regardless, metalloprotease genes like m41 ftsh are upregulated in DENV and ZIKV-infected *Ae. aegypti* (Sim et al., 2012; Etebari et al., 2017) suggesting *Wolbachia* may be utilizing host miRNAs to control a specific host gene essential for its growth which arboviruses also need. Conversely, *miR-2940* suppresses DNA methyltransferase (*AaDnmt2*) in *w*Melpop-CLA transinfected mosquitoes (Zhang et al., 2013). Some of the biological functions of *AaDnmt2* are host defense, genome stability, and lifespan regulation. Presence of DENV in *Ae. aegypti* without the endosymbiont produces higher levels of this gene. In this case, *Wolbachia* creates a cellular environment unconducive or antagonistic to the virus (Zhang et al., 2013).

Meanwhile, miRNAs can also have an effect on host autophagy, viral replication, and cellular transport. As an example, *w*MelPop-CLA in Aag2 cells triggers marked expression of *aae-miR-12* capable of suppressing two genes namely monocarboxylate transporter (*MCT1*) and DNA replication licensing factor (*MCM6*) (Osei-Amo et al., 2012). The exact role of these genes in *Ae. Aegypti* is unknown but existing studies in other insects reveal that *MCT1* is a key player in autophagy (Velentzas et al., 2018) whereas in DENV and ZIKV infection, these viruses take over the host’s autophagy response to evade the host immune defenses (Choi et al., 2018). One possibility that requires further investigations is that *Wolbachia* produces a miRNA that can suppress the activity of MCT1 and therefore, autophagy.

*Wolbachia* infection also induces the expression of *aae-miR-981* and in effect, downregulates importin β-4 in *w*MelPop-CLA infected Aag2. Reducing the activity of importin β-4 blocks AGO1 translocation to the nucleus (Hussain et al., 2013). There is no existing data that answers why hindering AGO1 translocation to the nucleus is advantageous for *Wolbachia’s* viral blocking. Nevertheless, importin β serves a different role in arboviral infection. Importin β binds to DENV and ZIKV non-structural proteins to assist in their nuclear migration for optimal replication as observed in *Ae. Albopictus* (Pryor et al., 2007; Ji and Luo, 2019). If this effect is the same for *Ae. aegypti* then the downregulation of importin β during *Wolbachia* infection may not only impair AGO1 translocation but also potentially block viral transcription.

Finally, *Wolbachia*-derived miRNAs that may contribute to viral blocking have also been reported. For instance, *WsRNA-46* found in *Wolbachia*-infected Ae*. aegypti* promotes dynein expression, a cytoskeletal protein important in cellular transport, mRNA localization, and movement of lipid droplets (Mayoral et al., 2014). In both *Wolbachia* and arboviruses, dynein is crucial for maintaining density and infection (Baldridge et al., 2014; Mayoral et al., 2014). Given that arboviruses also use the same cytoskeletal structure for their benefit, this represents an overlapping need for the same host cellular factors.

## Concluding Remarks

In recent years, *Wolbachia*-infected *Ae. aegypti* are being released to control medically important arboviruses (O’Neill et al., 2019; Indriani et al., 2020). The impact of *Wolbachia* on arboviral inhibition has been validated by looking into disease prevalence after mass release programs have been implemented (Indriani et al., 2020) and by simulating its effect computationally (Zhang and Lui, 2020). However, elucidating the exact cellular mechanisms that block off these viruses inside the mosquito cell is still underway. The two well-established hypotheses suggest that the transinfection of *Wolbachia* into *Ae. aegypti* induces an immune reaction that fights off the invading viruses and that the endosymbiont wins the competition for scarce resources (Kambris et al., 2010; Pan et al., 2012; Caragata et al., 2016). Based on the facts presented in this review, there could be another potential explanation as to how *Wolbachia* blocks a pathogen. It is likely that *Wolbachia* directly interferes with arboviruses. For instance, *Wolbachia* has been discovered to reduce host proteins that help viruses enter the cell (Lu et al., 2020). *Koh et al.* also demonstrated this antagonistic effect when dual infection of *Wolbachia* and DENV in *Ae. aegypti* resulted in a decrease in specific types of lipids that are normally upregulated when only the virus is present (Koh et al., 2020). More so, the endosymbiont enhances ROS production (Pan et al., 2012) and cellular regeneration (Taracena et al., 2018) that can directly harm an invading pathogen as well as prevent systemic viral infection, respectively. More interestingly, these antagonistic effects are consistent with the contrasting patterns in gene expression when either microbe are present or both (**Table 3**).

**Table 3:** Summary of interactions between *Ae. aegypti-*Arboviruses-*Wolbachia*.

Host cellular factor/mechanism	*Ae. aegypti* + virus	*Ae. aegypti* + *Wolbachia*	*Ae. aegypti +* virus + *Wolbachia*
Cytoskeleton	Upregulated to aid in cell transport and viral life cycle	Upregulated to maintain localization and density within the host	Downregulated to inhibit virus infection
Midgut barrier/Cellular regeneration	Earlier formation of a thicker PM	Activation of Delta/Notch and Cadherin to increase host fitness	Delta/Notch activation both for host fitness and regeneration of midgut epithelium
	Delayed regeneration of midgut epithelium *via* Delta/Notch		
	Upregulated proteolytic transcripts to break host’s midgut epithelium		
Metabolism	Increased cellular lipids/cholesterol for viral replication	Increase stored cholesterol with LD accumulation for maintaining density	Depletion of specific lipids that are needed by arboviruses
	Insulin/LD activate immunity		
Immunity/ROS	Downregulated immune-related transcripts Decrease ROS with antioxidant production	Upregulates immunity	Increase ROS to activate immunity. Releases antioxidants to maintain density

Wolbachia’s pathogen blocking is mediated by cellular and molecular changes that occur in different host cell processes. Individual infection of arboviruses compromises Ae. aegypti antiviral responses whereas Wolbachia infection in the same host strengthens it. Presence of both arbovirus and Wolbachia demonstrate antagonistic interference by the endosymbiont to block the invading pathogen.

Discovering the cellular mechanisms responsible for *Wolbachia*’s pathogen blocking effect remains challenging due to the complex relationship between three factors, namely the host, arboviruses, and *Wolbachia.* In terms of the host, *Ae. aegypti* colonies despite having the same strain can be highly divergent when reared in different laboratories and these laboratory-reared mosquitoes are less genetically variable compared to their field counterparts (Gloria-Soria et al., 2019). Susceptibility to viruses also vary depending on *Ae. aegypti* strain (Perrin et al., 2020) and possibly *Wolbachia* considering that some *Ae. aegypti* mosquitoes have been reported to carry natural infection. When it comes to arboviruses, DENV, ZIKV, and CHIKV may differ in the extent to which they can infect the host. *Wolbachia pipentis* also consists of several strains that may induce antiviral protection depending on the virus and the extent by which it alters host cellular mechanisms. Collectively, these make pathogen blocking mechanisms elusive.

Our understanding of *Wolbachia*’s pathogen blocking is mostly based on *Drosophila melanogaster* as a model organism highlighting the need to establish *Ae. aegypti* models. To determine if this vector control strategy is indeed sustainable, focusing on locations where mass release programs have been conducted could be a reliable indicator for effectiveness. *Ae. aegypti* mosquitoes can be collected from these sites and those *Wolbachia*-infected should be characterized using transcriptomic, proteomic, and metabolomic approaches. The first question that could be addressed is, what are the unique features found in field-collected, artificially *Wolbachia*-infected *Ae. aegypti* as opposed to the uninfected? This baseline information can then be used for succeeding *in vitro* approaches to answer the question, how are these unique genes, proteins, and metabolites affected when virus infection occurs in *Wolbachia-*infected cells? From this information, we can then focus on specific cellular mechanisms and validate how these are used/altered in virus-infected, *Wolbachia*-infected and *Wolbachia*+virus-infected *Ae. aegypti.* Most of the studies determined the role of a host cell mechanism by silencing genes under the said pathway. Alternatively, applying the CRISPR/Cas9 technology *in vitro* to ensure complete gene knockout may be a better approach. Taking into account natural infection, an *in vitro* model representing this (e.g. co-infection of isolated *Wolbachia* with stable *Wolbachia*-infected cell line prior the virus) must also be included in the experimental design. Finally, acknowledging the variable effects of multiple *Wolbachia* strains should prompt investigators to compare the cellular mechanisms each *Wolbachia* strain elicits for pathogen blocking and ask which mechanisms induce greater magnitude of viral inhibition. A targeted yet comprehensive view on the *Wolbachia* pathogen blocking mechanisms dissected using advanced approaches such as omics and gene editing will lead to efficient, sustainable and safe arbovirus control.

## Author Contributions

JR and KW conceptualized the review article. JR wrote and revised the manuscript with KW’s supervision. JR created the figures. YS and TC added concepts. KW, YS, TC, and MM reviewed and revised the initial drafts. All authors contributed to the article and approved the submitted version.

## Funding

The publication of this article is supported by Japan Society for the Promotion of Science (JSPS) Core-to-Core Program B. Asia-Africa Science Platforms (JPJSCCB20210006) and JSPS Grant-in-Aid Fund for the Promotion of Joint International Research (Fostering Joint International Research (B)) (19KK0107).

## Conflict of Interest

The authors declare that the research was conducted in the absence of any commercial or financial relationships that could be construed as a potential conflict of interest.

## Abbreviations

DENV, dengue virus; ZIKV, zika virus; CHIKV, chikungunya virus; LD, lipid droplet; Dpi, days post infection.
